# Advances in Antimicrobial Peptides: Mechanisms, Design Innovations, and Biomedical Potential

**DOI:** 10.3390/molecules30071529

**Published:** 2025-03-29

**Authors:** He Zhang, Jiaxun Lv, Zhili Ma, Junfeng Ma, Jing Chen

**Affiliations:** State Key Laboratory for Diagnosis and Treatment of Severe Zoonotic Infectious Diseases, Key Laboratory for Zoonosis Research of the Ministry of Education, School of Life Sciences, Jilin University, Changchun 130012, China; zhanghe8221@mails.jlu.edu.cn (H.Z.); 15022124223@163.com (J.L.); mazl22@mails.jlu.edu.cn (Z.M.)

**Keywords:** antimicrobial peptides, antimicrobial mechanisms, membrane disruption, synergistic antimicrobial effects, nanoparticle delivery, hydrogels, computer-aided design

## Abstract

This comprehensive review explores the advancements in the study of antimicrobial peptides (AMPs), highlighting their potential as promising alternatives to conventional antibiotics in the context of growing antibiotic resistance. AMPs are small molecular proteins found ubiquitously in nature, exhibiting broad-spectrum antimicrobial activity, including antibacterial, antiviral, and antifungal effects, and are vital components of the innate immune system. Due to their non-specific membrane-disrupting mechanism, AMPs are emerging as effective candidates for novel anti-infective agents. The integration of AMPs with biomaterials, such as nanoparticles, liposomes, polymers, and hydrogels, enhances their stability and efficacy while offering multifunctional therapeutic benefits. These combinations promote diverse antibacterial mechanisms, including membrane disruption, intracellular metabolic interference, cell wall modulation, and immune system activation. Despite challenges, such as toxicity, stability, and resistance, innovative strategies including computer-aided design and structural modification show promise in optimizing AMPs’ activity, targeting precision, and biocompatibility. The potential for AMPs in clinical applications remains highly promising, with significant opportunities for overcoming antimicrobial resistance through novel AMP-based therapeutic strategies.

## 1. Introduction

The increasing global issue of antibiotic resistance, driven in part by the misuse and overuse of antibiotics, is rendering many current treatments ineffective, potentially ushering in an era where existing antibiotics become obsolete [1,2]. Antimicrobial peptides (AMPs) have emerged as promising candidates for the next generation of antibiotics due to their unique non-specific mechanisms of action [3]. These small peptides, found across a wide range of organisms, offer a rapid innate immune response to microbial invasions, providing essential protection against bacterial, viral, and fungal pathogens [4,5,6]. Early research into AMPs primarily focused on their discovery and the evaluation of basic antimicrobial activities. The advent of genomics and proteomics in the early 2000s accelerated research, enabling the systematic study of AMP gene expression, regulatory pathways, and their roles in host defense [7,8].

In contrast to traditional antibiotics, which typically target a limited number of bacterial proteins, AMPs exhibit a diverse range of mechanisms. These include disruption of microbial cell membranes, interference with intracellular processes, modulation of cell wall synthesis, and the activation of immune responses, all of which reduce the likelihood of resistance development [9,10,11,12]. This multifaceted mode of action not only enhances their efficacy against drug-resistant pathogens but also allows for rapid antimicrobial responses and reduces the selective pressures that promote resistance. Additionally, AMPs have demonstrated a favorable safety profile, with negligible toxicity toward mammalian cells [13], thereby positioning them as promising candidates in the medical field. Recent innovations in AMP formulation have included the conjugation of these peptides with biomaterials such as metal nanoparticles, hydrogels, and liposomes. These conjugates enhance the stability, biocompatibility, and antibacterial efficacy of AMPs in vivo, opening new avenues for their clinical application. With the rapid advancements in computer-aided design, there has been growing interest in the development and optimization of AMPs to better meet therapeutic expectations [14]. As research progresses, AMPs are increasingly seen as a promising solution to the escalating challenge of antibiotic resistance.

**Figure 1:**
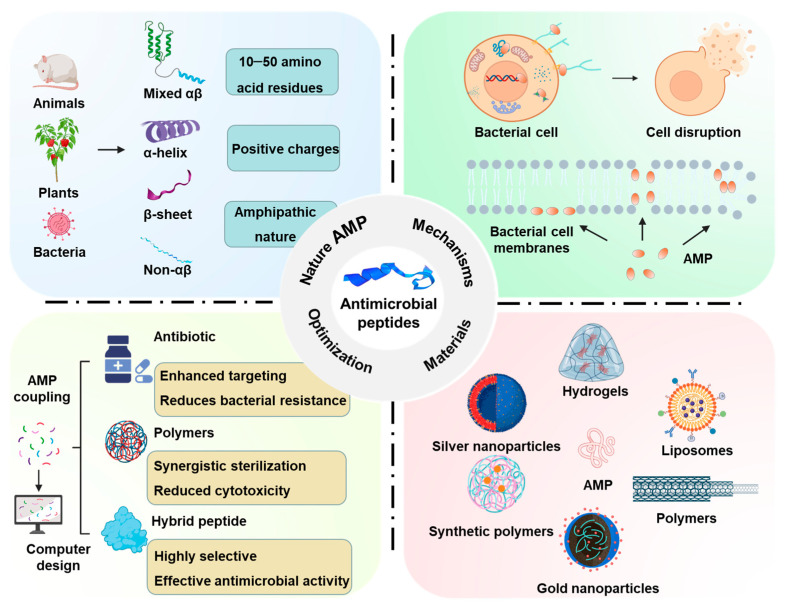
Overview of the content covered in review.

This review aims to provide a comprehensive overview of the classification, structure, mechanisms of action, and biomedical applications of AMPs (Figure 1). It will also explore the latest advances in the design and optimization of these peptides, highlighting the strategies employed to enhance their therapeutic potential. Finally, the challenges and opportunities associated with AMP development will be discussed, with an emphasis on their future role in addressing antibiotic resistance. By synthesizing current research, this review underscores the significance of AMPs as a promising class of therapeutics in the ongoing fight against multidrug-resistant pathogens.

## 2. Classification of AMPs

AMPs are typically small peptides consisting of 10–50 amino acid residues, and their molecular weight usually ranges from 1.5 to 6 kDa. The discovery of AMPs dates back to the early 20th century, when antibacterial properties were first identified in biological fluids such as serum [15]. However, it was not until the 1960s that AMPs were isolated from mammalian polymorphonuclear leukocytes, marking a major milestone in the study of these peptides [16]. With advances in molecular biology, the isolation and characterization of AMPs have been proliferated, expanding the known repertoire to include peptides like magainins from amphibian skin [17] and cecropins from insects [18]. Understanding the classification of AMPs based on their sources, structure, and molecular targets provides insight into their potential therapeutic applications.

### 2.1. Classification Based on Biological Sources

AMPs can be classified according to the biological sources from which they are derived. These sources include animals, plants, and microorganisms, and each source contributes to the diversity of AMP structures and mechanisms of action (Table 1) [19].

#### 2.1.1. Animal-Derived AMPs

The discovery of AMPs in animals marked a significant step in the study of AMPs. These peptides are a critical part of the innate immune system in many animals, playing roles in the skin, respiratory tract, digestive tract, and other mucosal surfaces. For instance, human cathelicidins such as LL-37 are potent AMPs found in human skin, providing a barrier against pathogen entry [20]. Cecropins from insects have also been studied extensively and are known for their ability to disrupt bacterial membranes [18]. Defensins, present in many mammals, also exhibit broad antimicrobial activity, especially against Gram-negative (G−) bacteria [21].

#### 2.1.2. Plant-Derived AMPs

Plants have evolved AMPs as part of their defense against herbivores and microbial pathogens. These peptides are often small, cysteine-rich, and function in various plant tissues such as seeds, leaves, and roots. Representative examples included thionins and lectins, which are known for their ability to defend against bacteria, fungi, and viruses [22].

Cyclotides are another class of plant-derived AMPs, characterized by their head-to-tail cyclized backbone and cystine knot motif, which confer exceptional stability against enzymatic degradation [23]. These structural features allow cyclotides to remain bioactive under harsh physiological conditions. Some cyclotides, such as kalata B1, have been reported to disrupt bacterial membranes, leading to cell lysis [24]. Their potent activity, combined with remarkable stability, makes them promising candidates for antimicrobial drug development.

#### 2.1.3. Microbial-Derived AMPs

Microbial-derived AMPs: Many microorganisms, including bacteria and fungi, produce AMPs to protect themselves against competing microbial species. These peptides are a part of their natural defense mechanism and have also been harnessed for therapeutic use. For instance, nisin A from *Lactococcus lactis* is a well-known AMP used as a food preservative, effective against Gram-positive (G+) bacteria [25].

**Table 1:** Natural AMPs: sources, mechanisms of action, amino acid sequences, and 3D structures.

Name	Source	Bactericidal Activity	Mechanism of Action	Amino Acid Sequence	3D Structure ^(a,b)^
LysAB2 P3 [26]	*Acinetobacter baumannii phage*	*A. baumannii*	Degradation of peptidoglycan cell wall and subsequent decomposition of bacterial cells	NPEKALEKLIAIQKAIKGMLNGWFTGVGFRRKR	α-helix, β-sheet ^a^
PK34 [27]	*Mycobacterium phage*	*Mycobacterium tuberculosis*	Inactivation of MAPK and PKB signaling reduces inflammatory cytokines secretion	LPRVIETKVHGREVTGLARNVSEENVDRLAKRWIK	α-helix, β-sheet ^a^
Human β-defensin 3 [28]	*Homo sapiens*	*Staphylococcus epidermidis*	Downregulation of genes responsible for biofilm formation	GIINTLQKYYCRVRGGRCAVLSCLPKEEQIGKCSTRGRKCCRRKK	α-helix, β-sheet ^a^
Microcin J25 [29]	*E. coli*	G− bacteria	Bind to RNA polymerase and inhibit the activity of RNA polymerase	GGAGHVPEYFVGIGTPISFYG	β-sheet ^a^
Satanin 1 [30]	*Scarabaeidae*	G− bacteria	Inhibits the release of pro-inflammatory cytokines such as tumor necrosis factor-α	RSKKWRKIEKRVKKIFEKTKEALPVIQGVATIVGAVGR	α-helix ^b^
Bac-7 [31]	*Bos taurus*	*E. coli*, *Salmonella typhimurium*	Inhibit 70S ribosome protein synthesis and DnaK activity	RRIRPRPPRLPRPRPRPFPRPGPRPRPRFPLPFP	β-sheet ^a^
LL-37 [32]	*Homo sapiens*	*Severe Acute Respiratory Syndrome Coronavirus-2*	Inhibit bacterial adhesion; disruption of cell signaling system	LLGDFFRKSKEKIGKEFKRIVQRIKDFLRNLVPRTES	α-helix ^a^
TO17 [33]	*Sciaenops ocellatus*	Infectious spleen and kidney necrosis virus	Induce degradation of genomic DNA and total RNA	KCRRRKVHGPMIRIRKK	β-sheet ^a^
Buforin 2 [34]	*Sphaenorhynchus lacteus*	*S. Aureus*, *E. coli*	Bind with nucleic acids and finally inhibit the synthesis of DNA, RNA, and proteins	TRSSRAGLQFPVGRVHRLLRK	α-helix ^a^
Pseudin-T2 [35]	*Pseudis paradoxa*	G− bacteria	Binds to DNA and forms pores on the cell membrane surface	LNALKKVFQKIHEAIKLI	α-helix ^a^
Andropin [36]	*Androctonus australis*	G+ bacteria	Reducing the tissue levels of pro-inflammatory cytokines to inhibit inflammation	VFIDILDKVENAIHNAAQVGIGFAKPFEKLINPK	α-helix ^b^
LactoferricinB [37]	*Bos taurus*	G+ bacteria, G− bacteria	Cause damage to the cell membrane	FKCRRWQWRMKKLGAPSITCVRRAF	β-sheet ^a^
Bactericidin B-3 [38]	*Manduca sexta*	G+ bacteria, G− bacteria	*	WNPFKELERAGQRVRDAIISAGPAVATVGQAAAIARG	α-helix ^b^
Bombinin H4 [39]	*Bombina variegata*	G+ bacteria, G− bacteria	Causes significant damage to the cell membrane, leading to protein leakage	ILGPVLGLVGSALGGLLKKI	α-helix ^a^
Maximin 5 [40]	*Bombina maxima*	G+ bacteria, G− bacteria	Membranolytic mechanisms promoted by anionic lipid	SIGAKILGGVKTFFKGALKELASTYLQ	α-helix ^a^
Brevinin-1 [41]	*Rana brevipoda porsa*	*S. aureus*, *Klebsiella Pneumoniae*	Lipopolysaccharide-neutralizing and anti-inflammatory activities	FLPVLAGIAAKVVPALFCKITKKC	α-helix, β-sheet ^b^
Esculentin-1 [42]	*Rana esculenta*	*E. coli*, *S. Aureus*, *P. aeruginosa*	Disrupts membrane integrity, hydrolyzes DNA, and activates pro-inflammatory cytokines	GIFSKLGRKKIKNLLISGLKNVGKEVGMDVVRTGIDIAGCKIKGEC	α-helix ^a^
Rugosin A [43]	*Rana rugosa*	*S. aureus*, *Bacillus subtilis*	Anti-inflammatory, promotes insulin secretion	GLLNTFKDWAISIAKGAGKGVLTTLSCKLDKSC	α-helix ^b^
Thanatin [11]	*Podisus maculiventris*	G+ bacteria, G− bacteria	Competes with Ca^2+^ and Mg^2+^ for lipopolysaccharide, disrupts membrane integrity, inhibits NDM-1 activity	GSKKPVPIIYCNRRTGKCQRM	β-sheet ^a^
AalCecA [44]	*Aedes albopictus*	G− bacteria	*	GGLKKLGKKLEGVGKRVFKASEKALPVAVGIKALG	α-helix ^b^
Cecropin [45]	*Antheraea pernyi*	G+ bacteria, G− bacteria	Penetrates cardiolipin-containing bilayers, causing lipid instability.	WNPFKELERAGQRVRDAIISAGPAVATVAQATALAK	α-helix ^b^
Melittin [46]	*Apis mellifera*	G+ bacteria, G− bacteria	Membrane permeabilization, ROS-mediated apoptosis, and (1,3)-β-D-glucan synthase inhibition	GIGAVLKVLTTGLPALISWIKRKRQQ	α-helix ^a^
Indolicidin [47]	*Bos taurus*	G+ bacteria, G− bacteria	Indole derivatives increase membrane permeability, disrupting osmotic balance and causing cell rupture	ILPWKWPWWPWRR	Non-αβ ^a^
Tritrpticin [48]	*Sus scrofa*	G+ bacteria, G− bacteria	Permeabilization of the cytoplasmic membrane	VRRFPWWWPFLRR	Non-αβ ^a^
BTD-1 [49]	*Papio anubis*	*E. coli*, *S. aureus*	Cell permeabilization, intracellular accumulation of reactive oxygen species	GFCRCVCRRGVCRCVCTR	β-sheet ^a^
Androctonin [50]	*Androctonus australis*	G+ bacteria, G− bacteria	High affinity for the postsynaptic acetylcholine receptor	RSVCRQIKICRRRGGCYYKCTNRPY	β-sheet ^a^
Dermaseptin-S1 [51]	*Phyllomedusa sauvagii*	G+ bacteria, G− bacteria	Decreases expression of hyphal wall protein 1 and aspartic proteases genes	ALWKTMLKKLGTMALHAGKAALGAAADTISQGTQ	α-helix ^a^
Pyrrhocoricin [52]	*Pyrrhocoris apterus*	G+ bacteria, G− bacteria	Inhibits the translation process in the protein synthesis system	VDKGSYLPRPTPPRPIYNRN	Non-αβ ^a^
Drosocin [53]	*Drosophila melanogaster*	G+ bacteria, G− bacteria	Inhibits protein synthesis by blocking 50S ribosomal subunit assembly	GKPRPYSPRPTSHPRPIRV	Non-αβ ^a^
Gomesin [54]	*Acanthoscurria gomesiana*	G+ bacteria, G− bacteria	L-type calcium channel influx, MAPK/ERK, PKC, and PI3K signaling, and ROS generation	QCRRLCYKQRCVTYCRGR	β-sheet ^a^
Enbocin [55]	*Bombyx mori*	G+ bacteria, G− bacteria	*	PWNIFKEIERAVARTRDAVISAGPAVRTVAAATSVAS	α-helix ^b^

^a^ 3D structure provided in reference; ^b^ 3D structure predicted by AlphaFold 3; * means that mechanism not yet discovered.

### 2.2. Classification Based on Three-Dimensional Structures

The three-dimensional (3D) structures of AMPs are crucial for their function, as these structures largely determine how AMPs interact with microbial membranes. The 3D structures of the currently explored AMPs can be determined using a variety of structural biology techniques, including X-ray crystallography, nuclear magnetic resonance (NMR) spectroscopy, circular dichroism (CD) spectroscopy, and more recently, the Alphafold 3 algorithm, which uses computational methods to predict protein structures. For more complex structures, 3D NMR [56] methods employing isotopically labeled peptides are indispensable, as demonstrated in the structure determination of human LL-37. Additionally, natural abundance heteronuclei 15N and 13C NMR [57] spectroscopy can provide further insights, particularly for short peptides rich in amino acids.

Due to their relatively small molecular weight, the 3D structures of AMPs are often influenced by environmental conditions, such as organic solvents, SDS, or DPC [58] The structural classification of AMPs is usually based on whether they form α-helices, β-sheets, or other configurations. These structural properties are essential for their antimicrobial activity.

#### 2.2.1. α-Helix Structure Peptides

AMPs with α-helical structures tend to insert directly into microbial membranes, where they form pores that lead to the leakage of cellular contents, causing bacterial cell death. For instance, melittin, from bee venom, is a well-known example of an α-helical AMP that causes cell lysis by forming pores in bacterial membranes [59]. Magainin 2, isolated from amphibian skin, also adopts an α-helical structure and disrupts bacterial membranes [60].

#### 2.2.2. β-Sheet Structure Peptides

β-sheet peptides are typically stabilized by disulfide bonds. These peptides are capable of penetrating microbial membranes and activating the immune system. For example, defensins are prominent β-sheet AMPs with antimicrobial and immune-modulatory effects [21], Thanatin, from insects, exhibits antimicrobial and immune-modulatory activity through β-sheet formation [61].

#### 2.2.3. αβ Structure Peptides

Peptides such as Psd1 [62], Heliomicin [63], and Phormicin [64] are highly stable and can disrupt bacterial biofilms or interfere with bacterial cell wall synthesis.

#### 2.2.4. Non-αβ Structure Peptides

Peptides such as Drosocin [65], nisin A [24], and mersacidin [66] can induce oxidative stress responses in bacteria, disrupting the oxidative-reductive equilibrium within bacterial cells, or interact with intracellular targets like nucleic acids, proteins, or organelles, thereby interfering with bacterial physiological functions.

### 2.3. Classification Based on Molecular Targets

AMPs can also be classified based on the specific molecular targets they interact with, which determines their mechanism of action.

#### 2.3.1. Cell-Penetrating Peptides

These peptides primarily interact with the bacterial cell membrane, causing disruption or pore formation. This leads to the leakage of cellular contents and ultimately bacterial death. Examples of cell surface-targeting peptides include ABP-CM4 [67] and bombesin [68], which are known for their effectiveness in disrupting bacterial cell membranes.

#### 2.3.2. Cell Interior-Targeting Peptides

In contrast to surface-targeting peptides, these AMPs exert their antimicrobial effects by penetrating the bacterial cell membrane and targeting intracellular processes. They can interfere with essential metabolic pathways or inhibit protein synthesis, thereby disrupting bacterial growth and function. Representative examples of cell interior-targeting peptides include BacA [69], which inhibits bacterial cell wall biosynthesis, and Apidecin [70], which interferes with bacterial protein synthesis.

## 3. Mechanisms of Action of Antimicrobial Peptides

**Figure 2:**
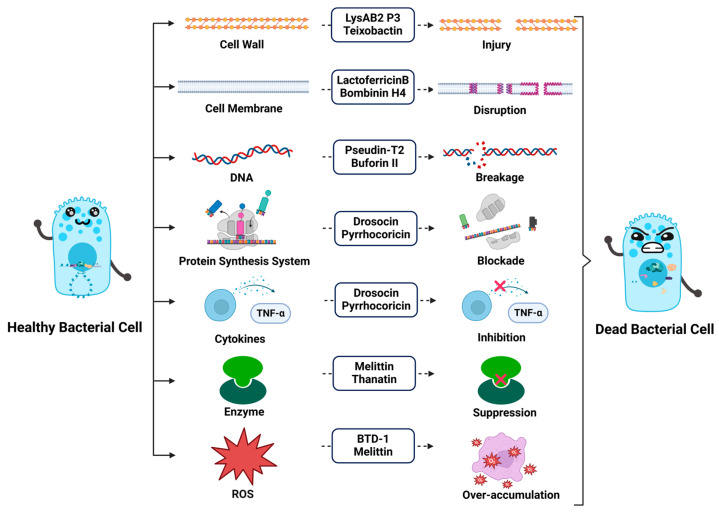
Antibacterial mechanisms of AMPs. AMPs exert their bactericidal effects through multiple mechanisms, including cell wall targeting, membrane targeting, and disruption of intracellular metabolic processes. Different AMPs act on distinct bacterial targets, and their ability to kill bacteria through diverse modes of action helps mitigate the development of resistance.

AMPs exert their effects through a variety of mechanisms, primarily by targeting and disrupting microbial structures that are vital for survival. Their ability to interact with different components of microbial cells—including the membrane, cell wall, DNA, protein synthesis system, cytokines, enzyme, and reactive oxygen species—enables them to combat a wide range of pathogens (Figure 2). The primary mode of action for most AMPs is the direct disruption of the cell membrane, as this structure is essential for maintaining cellular integrity. This section will delve into the mechanisms through which AMPs target the microbial membrane, cell wall, and intracellular machinery, leading to their destabilization and ultimately cell death.

### 3.1. Direct Disruption of Microbial Cell Membranes

One of the primary mechanisms by which AMPs exert their effects is by directly targeting and disrupting the microbial cell membrane. This membrane, which is essential for maintaining cellular integrity, serves as a critical point of attack for AMPs. The differences between bacterial and mammalian cell membranes enable AMPs to selectively recognize and disrupt microbial cells while sparing host cells [71].

For most AMPs, the structural distinctions between the outer membranes of G+ and G− bacteria lead to different modes of action. G+ bacteria possess negatively charged teichoic acids on their surface, facilitating the adsorption of cationic AMPs via electrostatic attraction. These peptides then penetrate the thick peptidoglycan layer and interact directly with the cytoplasmic membrane, where they bind to anionic phospholipids (e.g., phosphatidylglycerol) in the lipid bilayer [72]. In contrast, G− bacteria, despite having a thinner peptidoglycan layer, feature a complex outer membrane composed of lipopolysaccharides (LPS), phospholipids, and porins. AMPs must first traverse this barrier, often by binding to negatively charged LPS molecules and displacing divalent cations (e.g., Mg^2+^/Ca^2+^), thereby destabilizing the outer membrane [10].

Nevertheless, both types of bacteria share a fundamental similarity in their cytoplasmic membrane composition. Their membranes are enriched with phospholipids, phosphatidylglycerol, and cardiolipin—components characterized by predominantly anionic headgroups. These negatively charged moieties confer a strong electrostatic affinity for cationic AMPs, driving their targeted interaction with bacterial membranes [12]. AMPs then interact with the phospholipid bilayer via hydrophobic amino acid residues, often binding at the membrane surface and forming an initial membrane-bound complex [72]. Upon binding, AMPs undergo conformational changes, transitioning from a disordered or loose structure to a more ordered secondary structure, such as α-helices or β-sheets [73]. The insertion of AMPs into the membrane induces localized disturbances, forming transmembrane pores or channels that allow the non-selective passage of small molecules and ions [74]. As AMPs continue to insert and form pores, the integrity of the cell membrane is compromised, ultimately leading to membrane disruption [75].

**Figure 3:**
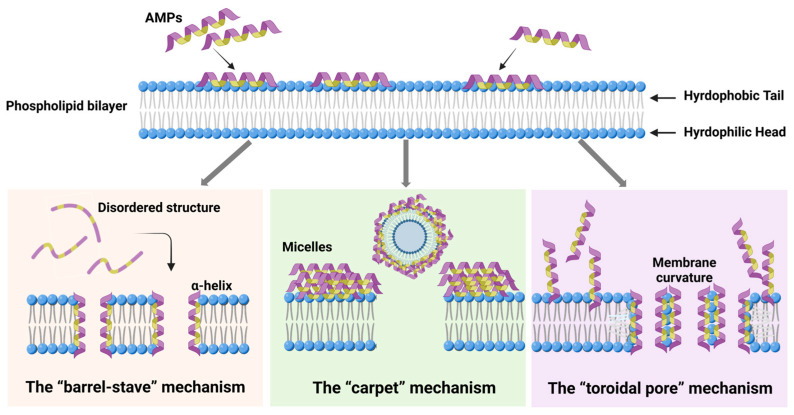
Three main membrane-disrupting mechanisms of AMPs, including the “barrel-stave”, “carpet”, and “toroidal pore” models. The hydrophilic and hydrophobic regions of the AMPs are represented in yellow and purple.

Current research indicates that AMPs typically disrupt bacterial cytoplasmic membranes through three mechanisms, as shown in Figure 3 [76]. The three main membrane-disrupting mechanisms are as follows:

(1) The “barrel-stave” mechanism: In this mechanism, AMPs insert into the phospholipid bilayer, undergoing lateral diffusion across the membrane. Once inserted, they undergo a conformational change, folding into an α-helix, which facilitates the formation of a barrel-like structure across the membrane. For example, Melittin, an AMP derived from bees, is a cationic linear peptide composed of 26 amino acid residues. Upon binding to the lipid bilayer, its structure changes from a disordered structure into an amphipathic α-helical structure [59]. In this way, the hydrophilic faces of the peptides orient toward the channel, while their hydrophobic faces interact with the interior of the phospholipid bilayer. This alignment induces the aggregation of lipids around the peptides, destabilizing the membrane and causing leakage of membrane components such as ions and small molecules, leading to a loss of membrane integrity and cell death [77,78,79].
(2) The “carpet” mechanism: Unlike the barrel-stave model, AMPs in the carpet mechanism do not penetrate the bilayer directly. Instead, they align parallel to the membrane surface, binding to the lipid head groups and covering the membrane like a carpet. As the concentration of AMPs increases, they accumulate on the membrane surface, and the peptides aggregate, forming a dense layer. This accumulation leads to a detergent-like effect, disrupting the membrane’s structural integrity by disturbing the packing of lipids. Eventually, this results in the formation of micelles, with the membrane destabilized and substances leaking out of the cell, compromising its physiological functions [80,81].
(3) The “toroidal pore” mechanism: This mechanism shares similarities with the carpet model; AMPs remain largely parallel to the membrane surface [82]. However, in the toroidal pore model, the peptides are oriented vertically and interact with the lipid heads at the membrane–water interface. This interaction induces a curvature in the membrane, leading to the formation of a pore with a water-filled core at the center. The hydrophilic residues of the peptides interact with the lipid heads, while the hydrophobic regions penetrate the bilayer. This structural change results in a distortion of the bilayer, where the lipid monolayer bends around the pore, forming a toroidal shape. The resulting pores allow the passage of ions and small molecules, disrupting the cell’s internal balance and contributing to cell death [83,84,85].

### 3.2. Impact on Cell Wall Formation

Recent works suggest that some AMPs are not randomly distributed on the surface of the cytoplasmic membrane, but rather aggregate at specific sites associated with structures involved in cell division and cell wall remodeling [86,87]. This targeted accumulation can interfere with these critical processes, leading to cell lysis.

Researchers found that the interaction between bacteria and GL13K-coated surfaces resulted in the disruption of the bacterial cell wall at the septum or polar regions, leaving behind shell-like structures or exposed protoplasts. GL13K, an AMP derived from human salivary protein BPIFA2, was found to kill 100% of *Streptococcus gordonii* at a concentration of 8 μg/mL [88]. Due to its covalent interactions, GL13K does not cross the bacterial cell membrane, suggesting that its bactericidal action occurs primarily through its impact on the bacterial cell wall. Additionally, it was observed that the L-enantiomer of GL13K enhances the autolysis of G+ bacteria, whereas its D-enantiomer completely inhibits this process [85]. The structures in the bacterial cell wall, such as teichoic acid and lipoteichoic acid, which carry negative charges, are capable of attracting positively charged AMPs, thereby facilitating their binding and enabling them to exert their antimicrobial functions [89].

### 3.3. Interference with Intracellular Metabolism

AMPs derived from mammals and invertebrates, especially those rich in arginine or proline, exhibit specific activity against G− bacteria. For instance, the synthetic peptide PR-26—a derivative of the proline-arginine-rich neutrophil AMP PR-39—exhibits a significantly lower MIC against G− pathogens compared to other analogues. It is supposed that the positively charged guanidinium group in arginine enhances the peptide’s electrostatic interaction with the negatively charged LPS on G− bacterial surfaces. In addition, the structural rigidity and conformational flexibility imparted by proline facilitate the peptide’s membrane insertion and disruption [90]. Following membrane interaction, these AMPs interact with receptors or docking molecules in the cytoplasmic membrane transport system through stereospecific mechanisms [91], or they are transported into the cell via endocytosis or other cellular processes [92,93,94]. Once inside, they interact with their intracellular targets, such as chaperone protein DnaK, or directly bind to nucleic acids, interfering with DNA replication and protein synthesis [93,95,96].

Each AMP has its unique mode of action, and interestingly, the same AMP can exhibit different mechanisms depending on its concentration. Notably, Bac-7 operates through two distinct mechanisms; (i) at concentrations close to its minimum inhibitory concentration (MIC), it relies on stereospecific uptake to bind intracellular targets, and (ii) at higher concentrations, it dissolves the membrane in a non-stereoselective manner, disrupting cellular integrity [97]. This multi-modal action is one of the key advantages of AMPs. Their ability to utilize different mechanisms reduces the likelihood of microorganisms developing resistance.

In addition to directly interacting with nucleic acids, AMPs can also target and inactivate nucleases involved in critical metabolic processes, including those responsible for DNA replication and repair [98]. Indole-3-acetonitrile, an AMP, specifically inhibits the activity of DNA topoisomerase I [99]. Topoisomerase I is an enzyme that alleviates the torsional strain generated during DNA replication by inducing transient single-strand breaks. By inhibiting this enzyme, indole-3-acetonitrile prevents DNA relaxation, which is a necessary step for the proper unwinding and progression of the replication fork. This interference disrupts DNA replication and ultimately impedes bacterial cell division.

### 3.4. Induction of Immune Responses

In addition to their bactericidal properties, certain AMPs also play a significant role in modulating immune responses [100,101,102,103]. For instance, AMPs derived from amphibians, such as frogs, have demonstrated potential therapeutic effects beyond antimicrobial activity. A notable example is the esculentin family, which includes Esculentin-1, Esculentin-1b, and Esculentin-2Cha. These peptides have been shown to promote insulin release, suggesting their role in glucose metabolism and potential benefits in managing conditions like diabetes [104,105].

Furthermore, the brevinin family of AMPs, such as Brevinin-1CBb, Brevinin-1Pa, Brevinin-1E, Brevinin-2GUb, and Brevinin-2EC, activate pathways involving cAMP-dependent protein kinase A (PKA) and C-dependent G-protein-sensitive pathways. These mechanisms are also related to Ras homolog family member G (RhoG) proteins, which play a key role in cellular signaling and immune modulation [106,107,108]. Bioinformatics analyses have suggested that increasing the net positive charge and reducing hydrophobicity in AMPs can enhance their ability to promote insulin secretion, further linking their antimicrobial and immunomodulatory functions [109].

On the other hand, some AMPs are involved in immune cell recruitment and inflammation regulation. Specifically, human defensins such as hBD2 and hBD3, as well as their murine orthologs (mBD4 and mBD14), can bind to chemokine receptors CCR2 and CCR6. This interaction promotes the migration of leukocytes to the site of infection, contributing to the body’s immune defense [100]. Additionally, human β-defensin DEFB126 is known for its high affinity for LPS, a major component of G− bacterial cell walls. DEFB126 can neutralize LPS and inhibit the induction of inflammatory cytokines such as IL-1β, IL-6, and TNF-α in macrophages [101]. This suggests that some AMPs not only directly neutralize microbial components but also modulate the host’s immune response to reduce excessive inflammation.

## 4. Biomedical Applications of Antimicrobial Peptides

AMPs have a wide range of sources, multiple bactericidal methods, and a lower likelihood of developing resistance, among many other advantages. These characteristics make them highly promising for clinical applications. Some AMPs have already been approved for therapeutic use. A prime example is gramicidin, which has been approved by the U.S. Food and Drug Administration (FDA) and exhibits significant activity against G+ bacteria while also inhibiting certain G− bacteria at high concentrations [110]. However, clinical trials have shown that infected tissues often exhibit high proteolytic activity, a defense mechanism against foreign invaders. Unfortunately, this proteolytic activity can also pose a barrier to the clinical effectiveness of AMPs [111]. In addition, protein-based drugs, including AMPs, typically suffer from poor stability and a short circulating half-life. To address these challenges, several innovative biomaterials have been developed to enhance the stability, bactericidal efficacy, target ability, and cell toxicity compatibility of AMPs. These developments provide a stronger foundation for the clinical use of AMPs.

### 4.1. Synthetic Polymers

Polymers are large molecules composed of repeating units of smaller molecules (monomers). There are many natural polymers, such as cellulose and chitosan, as well as numerous artificially synthesized ones, including poly (lactic-co-glycolic acid) (PLGA), polyethylene glycol (PEG), polyvinyl alcohol (PVA), and chitosan (CS). Current research indicates that polymers can be effectively combined with various antimicrobial drugs, including low-molecular-weight antibiotics and AMPs. These polymer systems not only exert antibacterial effects independently but also provide additional or even synergistic antibacterial effects when used in combination with antimicrobial drugs [112].

Numerous studies have demonstrated the advantages of using PLGA as a delivery system for AMPs. These advantages include the following: (i) the ability to achieve sustained release of AMPs at the target site, (ii) protection against degradation by hydrolases and proteases, and (iii) reduced cell toxicity in mammals [113]. As an example, a PLGA-based drug delivery system was developed to incorporate NZ2114, a derivative of plectasin, which exhibits excellent antibacterial activity against G+ bacteria, particularly *Staphylococcus aureus* and *Staphylococcus epidermidis*. The minimal inhibit concentration (MIC) of NZ2114 was found to be as low as 4 μg/mL. In vivo experiments demonstrated that the PLGA-based delivery system provided better antibacterial effects than NZ2114 alone, highlighting that PLGA can protect NZ2114 from the complex pH and enzyme environment in biofilms [114]. Deng and co-authors developed a novel nanomedicine by encapsulating thanatin, an AMP, within nanoparticles composed of PLGA and hyaluronic acid. This PLGA-based delivery system protected thanatin from degradation, reduced cytotoxicity, and significantly increased its serum half-life and bioavailability, thereby enhancing its efficacy against *Escherichia coli* (*E. coli*)-induced sepsis [115].

CS is a non-toxic, biocompatible, and biodegradable polymer that is suitable for various drug delivery applications, such as enhancing absorption, achieving controlled release, and improving bioadhesiveness. AMPs can be effectively loaded onto CS-based polymers, as these materials are nanoscale in size and can overcome in vivo barriers, thereby increasing permeability [116]. The FDA has classified CS as a generally recognized as safe (GRAS) material for wound dressings [117]. Rishi et al. developed a nano-embedding system composed of CS and tripolyphosphate to load cryptdin-2. Cryptdin-2 was attached both to the surface and the interior of the CS matrix, achieving a release time of up to thirty minutes. CS, which is soluble at acidic pH, successfully delivered cryptdin-2 to the intestinal wall in its biologically active form after oral administration in animal studies [118]. Additionally, Layer-by-layer technique was employed to synthesize hollow nanocapsules made from CS and alginate. By encapsulating glycomacropeptide (GMP) in the third layer, the system prevented immediate release and provided a longer delivery time to the site of action [119]. To enhance Buforin I’s resistance to external environments and control its release, it was encapsulated into chitosan/polyethylene oxide nanofibers. Experimental results showed that at a concentration of 20 × minimum bactericidal concentration (MBC), the performance was significantly superior to that of currently available antibiotics, and it exhibited excellent tensile strength [120]. These studies underscore the broad potential of CS nanoparticles as effective carriers for therapeutic AMPs.

### 4.2. Liposomes

Liposomes are nanoscale, hollow spherical vesicles composed of one or more concentric lipid bilayers, typically made from phospholipids and cholesterol. Due to the amphipathic nature of phospholipids, liposomes self-assemble into lipid nanoparticles (LNPs) [121]. LNPs possess excellent biocompatibility and safety because of their natural lipid components [122]. Moreover, the amphipathic property of phospholipids allows these liposomes to encapsulate hydrophobic AMPs within the lipid bilayer and hydrophilic AMPs in the aqueous core. This encapsulation helps protect the cargo from degradation in vivo [123]. Additionally, liposomes are highly customized; their size, lipid composition, and surface modifications can be adjusted to alter their physicochemical properties. Notably, variations in lipid composition significantly influence liposome packing, fluidity, and charge, which in turn affect stability and encapsulation efficiency. These modifications can reduce the degradation resistance of AMPs, enhance their controlled-release capability, and increase their affinity for specific targets [124].

For instance, Herrera et al. developed a formulation to deliver active Thuricin CD through the gastrointestinal tract and locally to the colon. The researchers prepared liposomes composed of L-α-phosphatidylcholine (soy, HSPC) and 1,2-Dipalmitoyl-sn-glycero-3-phosphoglycerol (DPPG), encapsulating Thuricin CD in anionic liposomes. Their study found that Thuricin CD remained active after exposure to pepsin in gastric and intestinal juices. Furthermore, the liposome-encapsulated Thuricin CD induced significant morphological changes, puncturing, and leakage of cellular contents in *Listeria monocytogenes* (*L. monocytogenes*), which were not observed in other samples. This suggests that liposome encapsulation not only enhances the stability of AMPs in challenging environments but also significantly increases their antimicrobial efficacy by maintaining their biological activity. The liposome suspension containing Thuricin CD was also stable at room temperature for over 21 days and at 4 °C for over 60 days, while remaining non-toxic to intestinal epithelial cells [125].

Likewise, encapsulating the AMP LL-37 within glyceryl monooleate (GMO) LNPs has been shown to significantly enhance antibacterial activity against G− bacteria, particularly those with LPS-containing surfaces. The presence of LPS also stabilized the α-helicity structure of LL-37, enhancing its antimicrobial efficacy [126]. In another study, a cholesterol-containing vesicle system was designed to deliver AMP JR2KC, which facilitated rapid and effective membrane disruption through localized peptide concentration in cholesterol-rich regions [127]. These studies highlight the potential of lipid-based carriers to enhance the efficacy of AMPs by promoting specific membrane interactions.

In a different approach, DP7-C-modified azithromycin (AZT)-loaded liposomes were used to treat methicillin-resistant *Staphylococcus aureus* (MRSA) infections. The modified liposomes provided synergistic antibacterial effects, demonstrating the potential of lipid-AMP complexes in improving the therapeutic performance of traditional antibiotics [128]. Additionally, conjugating AMP IG-25 to a carboxylic acid-terminated polymerized liposome results in an 18-fold increase in efficacy compared to LL-37, without an increase in cell toxicity, further emphasizing the potential of liposomes as a delivery vehicle to enhance AMP activity [129].

These studies collectively underline the versatility of liposome-based systems in enhancing the stability, targeting, and antimicrobial effectiveness of AMPs. By modifying the lipid composition and incorporating specific structural features, liposomes can significantly improve the pharmacokinetics and therapeutic potential of AMPs, making them a promising tool for clinical applications.

### 4.3. Hydrogels

Hydrogels, which are hydrophilic polymers with a three-dimensional porous structure, are formed by crosslinking hydrated polymers through physical or covalent interactions. They offer several advantages, including high water swelling capacity, oxygen permeability, excellent biocompatibility, and efficient drug loading and release properties [130]. These features make hydrogels ideal carriers for AMPs, improving their efficacy while reducing cytotoxicity [131]. The physicochemical properties of hydrogels, such as their swelling behavior and release profiles, largely depend on the crosslinking method. Physically crosslinked hydrogels rely on non-covalent interactions like hydrogen bonds, while chemically crosslinked hydrogels utilize covalent bonds and often require crosslinking agents like glutaraldehyde, genipin, or dopamine [132,133].

AMPs generally display disordered in aqueous solutions. However, certain engineered AMPs can form well-defined nanostructures through self-assembly, typically driven by hydrophobic interactions [75,134,135,136]. Despite this, their inability to spontaneously crosslink or entangle often prevents them from forming stable hydrogels on their own [137]. To address this, recent studies have focused on combining AMPs with other substances to promote hydrogel formation. For instance, Bai and colleagues demonstrated that the AMP A9K2 could form micelles and short nanorods in solution, which upon exposure to fetal bovine serum (FBS) or plasma amine oxidase (PAO) transformed into a hydrogel with excellent antibacterial activity against both G− and G+ bacteria. Notably, this hydrogel exhibited minimal cytotoxicity to mammalian cells, highlighting its potential for therapeutic use [138]. Similarly, a hydrogel composed of oxidized dextran (ODEX), AMP-modified hyaluronic acid (HA-AMP), and platelet-rich plasma (PRP) has been developed under physiological conditions. This hydrogel exhibited significant antibacterial activity against *Staphylococcus aureus* and *Pseudomonas aeruginosa* (*P. aeruginosa*) and inhibited pro-inflammatory cytokines (TNF-α, IL-1β, and IL-6) while promoting anti-inflammatory factors’ production, indicating its potential for treating infections and modulating inflammation [139]. Additionally, a composite hydrogel (HAMA/t-GL13K) fabricated through photo-crosslinking exhibited broad-spectrum antibacterial and anti-inflammatory properties by inhibiting the expression of inflammatory factors and scavenging ROS. Their results suggested that such hydrogels may offer potential for clinical applications in treating microbial infections and promoting wound healing [140].

In addition to these formulations, other studies have explored the incorporation of AMPs into hydrogels for specific clinical applications. As an example, a hydrogel complex incorporating the AMP HX-12C has been synthesized, exhibiting excellent mechanical and high antibacterial efficacy. This dressing not only promoted wound healing but also stimulated the collagen deposition, which is essential for tissue repair [141]. Furthermore, an O-CMCS/SAP hydrogel loaded with the novel AMP mel-d1, derived from melittin, has been developed, showing enhanced wound closure and skin regeneration in an *E. coli* infection model [142]. Furthermore, an innovative strategy has been introduced to embed two AMPs, i.e., WR (sequence: WRWRWR-NH_2_) or Bac2A (sequence: RLARIVVIRVAR-NH_2_), into hydrogels using methyl sulfonate betaine ester (SBMA) and acrylic acid (AAc) as hydrogel monomers. The resulting hydrogel demonstrated excellent antibacterial and anti-adhesive properties against both G+ and G− bacteria. Moreover, the hydrogel coating effectively protected the AMPs’ long-term bioactivity while maintaining the biocompatibility and antibacterial ability of the system [143].

These studies collectively underline the versatility of AMP-loaded hydrogels in treating a variety of infections and enhancing wound healing. By combining the natural antibacterial properties of AMPs with the unique physical properties of hydrogels, these systems can offer controlled, localized drug delivery while minimizing side effects. However, challenges remain in optimizing the release kinetics and long-term stability of these hydrogel systems, as well as ensuring that AMPs maintain their bioactivity in complex physiological environments. Future studies should focus on developing novel materials, optimizing the design of AMP loading systems, and investigating the safety and efficacy of AMP hydrogels in clinical settings to ensure broader therapeutic use.

### 4.4. Metal Nanoparticles

Metal-based nanoparticles (MNPs), such as gold nanoparticles (AuNPs) and silver nanoparticles (AgNPs), are gaining significant attention in the field of biomedical applications due to their unique physicochemical properties, including excellent biocompatibility, optical activity, conductivity, stability, and heat resistance [144]. These nanoparticles are highly versatile, as they can be easily modified into various bio-conjugates, thus offering multiple binding sites for AMPs through non-covalent interactions, such as hydrogen bonding, ionic interactions, and van der Waals forces [145]. Although non-covalent bonds are generally weak, their multivalent nature enhances the stability and delivery efficiency of AMP-loaded MNPs, which is particularly useful for the controlled release of AMPs to target tissues and organs in vivo [146]. One of the key advantages of MNPs is their non-biological nature, which allows them to evade immune recognition, a critical factor in therapeutic applications. Additionally, MNPs can be engineered to respond to external stimuli, such as magnetic fields, and exhibit localized surface plasmon resonance effects, which can further enhance their targeting capabilities and therapeutic effectiveness [147,148]. These features, combined with their ability to stabilize and protect AMPs, make MNPs an ideal choice for optimizing AMP delivery systems.

AuNPs are particularly well-suited for AMP delivery due to their excellent biocompatibility, antioxidant properties, and ability to catalyze reactions in vivo [149]. AuNPs can promote oxidative stress within bacterial cells by catalyzing the decomposition of superoxide radicals, which leads to bacterial cell death. Furthermore, AuNPs disrupt bacterial cell membranes by binding irreversibly thiol groups in bacterial proteins, such as NADH dehydrogenase, which in turn inhibits essential bacterial processes like respiration [150]. Researchers have effectively conjugated AMPs to AuNPs to leverage both the antibacterial activity of the nanoparticles and the therapeutic properties of the peptides. For instance, Park et al. demonstrated that the histidine-tagged AMP Lys AB2 P3-His, conjugated with AuNPs, significantly inhibited *Acinetobacter baumannii* (*A. baumannii*) infection in mice, with prolonged survival times following intraperitoneal injection [151]. Similarly, Chowdhury and colleagues conjugated the AMP VG16KRKP (VARGWKRKCPLFGKGG) to AuNPs, showing strong bacterial lysis activity, particularly against *Salmonella typhi* (*S. Typhi*) [152]. This conjugate effectively targeted both intracellular and extracellular bacteria, without cytotoxicity to eukaryotic cells. In addition, the utilization of AuNPs to prolong the bioactivity of the AMP LL37 was also proved to enhance wound-healing properties in vivo. The LL37-AuNPs conjugates exhibited better performance than free LL37 in accelerating wound closure, suggesting that AuNPs can not only enhance the antimicrobial activity of AMPs but also promote tissue regeneration [153].

AgNPs are widely known for their strong antibacterial properties. These properties arise from two main mechanisms: their ability to directly interact with bacterial membranes, leading to contact killing, and their ion-mediated effects, which induce oxidative stress within bacterial cells [154,155]. Pal and co-authors conjugated the cationic peptide Odorranain-A-OA1 (OA1) with AgNPs (10 nm), significantly improving the stability of the AMP and reducing toxicity to mammalian cells [156]. Likewise, Li et al. synthesized AgNPs@AMP complexes using the AMP (LLRR)3. Their study demonstrated that these nanoparticle-AMP conjugates not only enhanced the stability of AgNPs in aqueous solutions but also significantly boosted their antibacterial efficacy against *E. coli* and *Staphylococcus aureus*. The AgNP@AMP complex effectively disrupted bacterial membranes, leading to leakage of cellular contents, which further amplified their antibacterial activity [157].

These studies underscore the promising potential of combining AMPs with various biomaterials, which not only enhances their antibacterial activity but also improves their stability, biocompatibility, and overall therapeutic potential. The integration of AMPs with lipid carriers, nanomaterials, or hydrogels has led to the development of multifunctional systems capable of targeted delivery, controlled release, and enhanced tissue penetration. These combinations have proven effective in diverse clinical applications, such as treating infections, inflammatory diseases, and promoting wound healing.

### 4.5. Combination with Multiple Biomaterials

**Figure 4:**
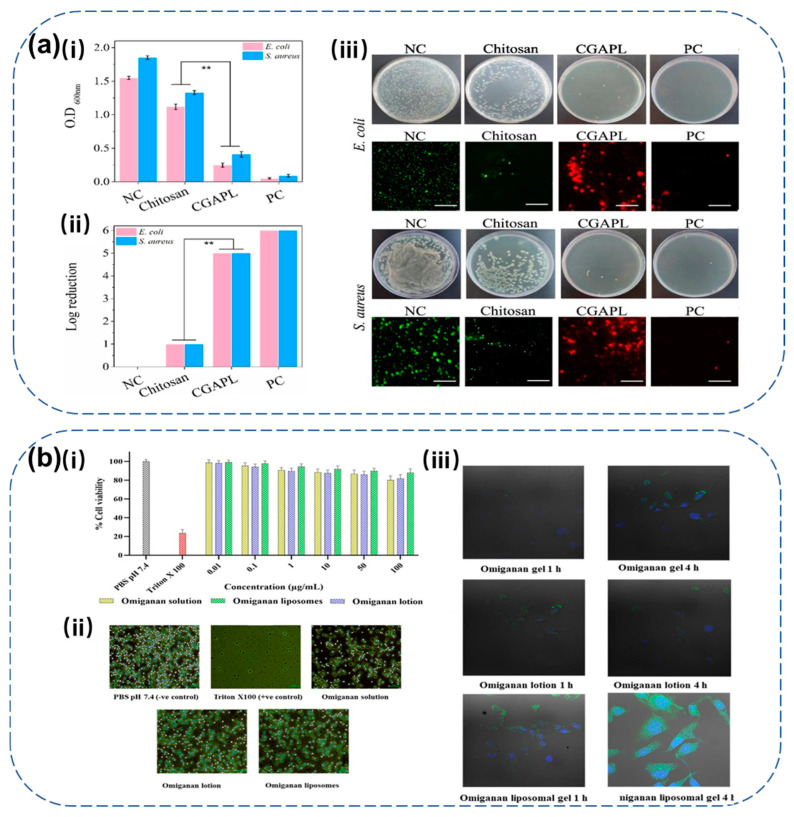
Two distinct approaches to enhancing the efficacy of AMPs in combination with biomaterials. (**a**) The integration of AMPs with AgNPs and hydrogels to form the nanobiocomposite hydrogel CGAPL. The antibacterial properties of chitosan and CGAPL were assessed against *E. coli* and *Staphylococcus aureus* through turbidimetry (**i**) and plate-counting assays (**ii**). Representative agar plate images and fluorescence micrographs (**iii**) show enhanced antibacterial action by CGAPL, highlighting its superior efficacy, ** *p* value < 0.006. Reproduced with permission from ref. [158]. Copyright 2023, Elsevier. (**b**) The combination of AMPs with liposomes and hydrogels, focusing on Omiganan formulations. In vitro results (**i**) demonstrate cell viability in 3T3 fibroblast cells, (**ii**) show cellular uptake of Omiganan formulations, and (**iii**) assess hemocompatibility. Reproduced with permission from ref. [161]. Copyright 2022, Elsevier.

The integration of AMPs with nanomaterials proves highly effective in wound-healing applications. One approach involved conjugating AMPs with graphene-AgNPs and embedding the resulting conjugate into chitosan hydrogels to create nanocomposite films (Figure 4a). The incorporation of graphene enhanced the mechanical properties of the hydrogel, providing the structural stability necessary for a functional wound dressing. The antibacterial AgNPs prevented bacterial growth, while AMP ε-poly-L-lysine added hydrophilicity, improving the stability, flexibility, and biocompatibility of the dressing. This combination demonstrated excellent potential for wound healing, showing both mechanical and antibacterial benefits [158]. Similarly, an antibacterial skin coating was developed for acne treatment by integrating AMP HHC36 with AgNPs conjugates in a carboxymethyl chitosan/sodium alginate (CMCS/SA) hydrogel. This composite hydrogel exhibited superior antibacterial performance compared to using the AMP or AgNPs alone, highlighting the potential of AMP–metal nanoparticle combinations in acne therapy [159].

In the same way, an antibacterial skin coating was developed for acne treatment by integrating AMP HHC36 with AgNPs conjugates in a carboxymethyl chitosan/sodium alginate (CMCS/SA) hydrogel. This composite hydrogel exhibited significantly enhanced antibacterial efficacy compared to the use of either AMP or AgNPs alone, underscoring the potential of AMP-metal nanoparticle formulations in dermatological applications.

Integrating the optimization of antimicrobial peptides (AMPs) with the strategic use of carrier systems is also an effective strategy to maximize their antibacterial potency. For instance, Cantor and colleagues explored two approaches: (i) structural modification of AMPs and (ii) incorporating the modified peptides into lipid nanocarriers. By coating liposomes with the polymer Eudragit E-100, the antibacterial activity of the two modified peptides against *L. monocytogenes* was significantly enhanced—2083-fold and 1562-fold, respectively [160]. Similarly, encapsulating Omiganan, an AMP, within liposomes using reverse-phase evaporation technology and incorporating them into Carbopol 934P hydrogel not only improves the encapsulation efficiency (72%) and loading efficiency (7.8%) but also facilitates controlled release and enhanced permeability compared to traditional formulations (Figure 4b). The approach resulted in a significant reduction in pro-inflammatory cytokines and improved the condition of atopic dermatitis and psoriasis lesions, demonstrating the therapeutic potential of AMP-loaded liposomes in inflammatory skin diseases [161].

These studies underscore the promise of combining AMPs with various biomaterials, not only enhancing their antibacterial activity but also improving their stability, biocompatibility, and therapeutic potential. By integrating AMPs with lipid carriers, nanomaterials, or hydrogels, researchers have been able to create multifunctional systems capable of targeted delivery, controlled release, and enhanced tissue penetration. This combination approach has opened valuable avenues for clinical applications, particularly in treating infections, inflammatory diseases, and promoting wound healing.

Despite the clear advantages, several challenges remain in the development of AMP–biomaterial combinations. One significant issue is the scalability of synthesizing these composite systems while maintaining consistent quality and efficacy. Furthermore, the stability of these systems in vivo, particularly their long-term effects, remains a critical consideration for clinical translation. There is also a need to refine the balance between antimicrobial activity and cytotoxicity, ensuring that AMP–biomaterial systems are both effective against pathogens and safe for human use. Moving forward, research should focus on optimizing the interactions between AMPs and biomaterials, enhancing their stability, biocompatibility, and targeting specificity, to ensure their successful clinical applications.

## 5. Design and Optimization of Antimicrobial Peptides

Although AMPs are less likely to develop resistance, certain mechanisms of resistance have already emerged in bacteria [162]. If these challenges are not addressed, we may face similar issues as with antibiotics, necessitating the search for new alternatives. To mitigate this, strategies like combining AMPs with antibiotics [163,164], as well as computer-aided design and structural optimization methods, have shown promise in improving the efficacy, stability, and safety of AMPs for clinical use.

### 5.1. Structure-Based Optimization of Antimicrobial Peptides

Natural AMPs often suffer from structural instability and sensitivity to physiological conditions, which limits their clinical application. Therefore, researchers have employed various structural optimization strategies to enhance their stability, antimicrobial efficacy, and therapeutic potential [165].

#### 5.1.1. Peptide Truncation and Amino Acid Substitution

By truncating the peptide chain or substituting amino acids, the stability and antimicrobial activity of AMPs can be significantly improved. Notably, Ye et al. obtained a truncated AMP (NCM4) from the green sea turtle’s Cm-CATH2, which exhibited enhanced antibacterial, anti-inflammatory and anti-biofilm properties with minimal cytotoxicity. Further ornithine substitution led to oNCM, which showed better stability without compromising antibacterial potency [166]. Likewise, KR-12, a truncated human host-defense peptide LL-37, was designed to a scaffold-cyclized dimer through disulfide, which showed improved stability under physiological conditions and retained strong antibacterial activities [167].

#### 5.1.2. Cyclization and Modifications

Cyclization via disulfide bonds or introducing helical structures can significantly enhance the stability and activity of AMPs. For example, crabrolin-TR, a modified AMP obtained from the venom of the European hornet, demonstrated enhanced antibacterial activity against *P. aeruginosa* [168]. Similarly, [Lys4,19,Leu20]R2AW(1-22)-NH_2_, a modified version of the helical-loop peptide ranatuerin-2-AW (R2AW) from the skin secretions of the Wuyi torrent frog, exhibited improved antimicrobial and anticancer activities due to enhanced cationicity and hydrophobicity [169].

These structural optimization strategies have paved the way for the development of AMPs with greater stability, antimicrobial potency, and reduced toxicity, which are crucial for their clinical applications.

### 5.2. Computer-Aided Design of Antimicrobial Peptides

Traditional methods of AMP development, including isolation from natural sources and chemical synthesis, are time-consuming and costly. With the rapid advancement of computer technology and bioinformatics, researchers can now design new AMPs using computer-aided design (CAD) techniques, significantly speeding up the discovery and optimization process (Table 2). These methods not only significantly shorten the drug-development cycle but also enhance the accuracy and efficiency of AMP design.

The computer prediction methods can be generally categorized into five categories, including AMPs (computer models predict short peptide sequences with antimicrobial activity), AMP precursors (predicting AMP precursor sequences, which are inactive forms of AMPs that can be processed into active peptides by specific enzymes in vivo), AMPs and precursors (simultaneously optimizing both AMPs and their precursors to enhance their functionality and activity), processing enzymes (predicting and designing enzymes that are responsible for processing AMP precursors into their active forms), and environmental factors (considering the interactions between AMPs and their environment (such as pH, salt concentration, and temperature), which helps in designing more stable AMPs under physiological conditions) (Table 3). These methods are supported by large AMP databases such as APD3 (AMP Database) [170] and AMPSphere [14], which include thousands of AMP sequences and their biological data. Researchers can search these databases for existing active peptides, which can be used to predict new sequences and characteristics of AMPs. APD3 currently arranges AMPs in the following manner: (1) natural AMPs; (2) AMPs with known amino acid sequences; (3) AMPs with known activities (e.g., MIC < 100 μM); and (4) peptides with fewer than 100 amino acids [170]. This organization allows for a more convenient and efficient search of AMPs, making it easier for researchers to find peptides with desired properties. Additionally, there are other tools for screening and identifying AMPs, such as dbAMP [171] and Ensemble-AMPPred [172], which further support the discovery of new peptides by providing access to a wide array of sequence and activity data (Table 4).

Computer predictions not only help efficiently identify potential AMP sequences but also allow simulations of how environmental factors affect AMP activity. For instance, using algorithms like Support Vector Machines (SVM), researchers can screen AMP sequences from the database and simulate their stability and antimicrobial activity under different pH values and temperatures. Pleurocidin, an AMP discovered using this method, demonstrated strong antibacterial potential in computer simulations and subsequent experimental validation [173]. Moreover, DP7, a computer-designed AMP, has been shown to effectively combat multidrug-resistant (MDR) *P. aeruginosa* and related infections, demonstrating strong antimicrobial activity in laboratory tests [174]. Alternatively, the Joker algorithm can also be used to design AMP sequences [175]. For instance, EcDBS1R6, originally a peptide derived from the signal peptide sequence of *E. coli*, was modified into an AMP through the Joker algorithm [176]. The advantage of computational design lies in its ability to generate AMP sequences with enhanced antimicrobial activity while minimizing unwanted toxicity or instability during synthesis.

**Table 2:** Artificially synthesized AMPs.

ID	Amino Acid Sequence	Activity	Target Site	3D Structure
Magainin-2 [F16W]	GIGKFLHSAKKFGKAWVGEIMNS	G+ bacteria, G− bacteria, Mammalian Cell	Lipid Bilayer	β-sheet
Dermaseptin S4 (1–13) AMD [M4K]	ALWKTLLKKVLKA	G+ bacteria, G− bacteria, Parasite, Fungus, Mammalian Cell	Lipid Bilayer	β-sheet
Dermaseptin S4 (1–16) [M4K]	ALWKTLLKKVLKAAAK	G+ bacteria, G− bacteria, Virus, Parasite, Fungus, Mammalian Cell	Lipid Bilayer, Virus entry	β-sheet
Dermaseptin S4 [M4K] [N20K]	ALWKTLLKKVLKAAAKAALKAVLVGANA	G+ bacteria, G− bacteria, Virus, Parasite, Cancer, Fungus, Mammalian Cell, Biofilm	Lipid Bilayer, Virus entry	β-sheet
Dermaseptin S4 (5–15) AMD	TLLKKVLKAAA	G+ bacteria, G− bacteria	Lipid Bilayer	β-sheet
Dermaseptin S4 (4–15) AMD [M4K]	KTLLKKVLKAAA	G+ bacteria, G− bacteria	Lipid Bilayer	β-sheet
Magainin-2 [S8K,A9V,K11S,A15S,F16W,V17I]	GIGHFLHKVKSFGKSWIGEIMNS	G+ bacteria, G− bacteria, Mammalian Cell	Lipid Bilayer	β-sheet
Magainin-2 [L6G,H7K,S8A,K10A,K11H,A15K,F16W]	GIAKFGKAAAHFGKKWVGELMNS	G+ bacteria, G− bacteria, Mammalian Cell	Lipid Bilayer	β-sheet
CAP7 (1–20) [L6K,I13K]	GLRKRKRKFRNKKKEKLKKI	G+ bacteria, G− bacteria	Lipid Bilayer	β-sheet
CAP7 (1–20) [R5A,K16A]	GLRKALRKFRNKIKEALKKI	G+ bacteria, G− bacteria	Lipid Bilayer	β-sheet
LL-37 fragment KR-12	KRIVQRIKDFLR	G+ bacteria, G− bacteria, Virus, Cancer, Fungus, Mammalian Cell, Biofilm	Lipid Bilayer, Virus replication	β-sheet
LL-37 (13–32) [I13G,G14I,E16Q]	GIKQFKRIVQRIKDFLRNLV	Virus, Mammalian Cell	Virus replication	β-sheet
Tritrpticin [V1R,W7VC]-R	RRRFPWVCWPFLRRR	Fungus	Lipid Bilayer	β-sheet
Cathelicidin-6 (1–15)	GRFKRFRKKFKKLFK	Virus, Mammalian Cell	Virus replication	β-sheet
Cathelicidin-6 (1–18) [F6I,F10L, L17I]	GRFKRIRKKLKKLFKKIS	Virus, Mammalian Cell	Virus replication	β-sheet
CP26, MBI 26	KWKSFIKKLTSAAKKVVTTAKPLISS	G+ bacteria, G− bacteria, Fungus, Mammalian Cell	Lipid Bilayer	β-sheet
CEME	KWKLFKKIGIGAVLKVLTTGLPALIS	G+ bacteria, G− bacteria, Fungus	Lipid Bilayer	β-sheet
CEMA, MBI-28	KWKLFKKIGIGAVLKVLTTGLPALKLTK	G+ bacteria, G− bacteria, Mammalian Cell	Lipid Bilayer	β-sheet
CP29, MBI 29	KWKSFIKKLTTAVKKVLTTGLPALIS	G+ bacteria, G− bacteria, Fungus, Mammalian Cell	Lipid Bilayer	β-sheet
BP100	KKLFKKILKYL	G+ bacteria, G− bacteria, Cancer, Fungus, Mammalian Cell	Lipid Bilayer	β-sheet
P18	KWKLFKKIPKFLHLAKKF	G+, G−, Cancer, Fungus, Mammalian Cell	Lipid Bilayer	β-sheet
Loop region of human lactoferricin	FQWQRNMRKVRGPPVS	G+ bacteria, G− bacteria	Lipid Bilayer	β-sheet
[RW]5	RWRWRWRWRW	G+ bacteria, G− bacteria, Fungus, Mammalian Cell	Lipid Bilayer	β-sheet
Amphipathic-1l, K6L9	LKLLKKLLKKLLKLL	G+ bacteria, G− bacteria, Mammalian Cell	Lipid Bilayer	β-sheet
(RW)3	RWRWRW	G+, G−, Cancer, Fungus, Mammalian Cell	Lipid Bilayer	β-sheet
B-38	IKQLLHFFQRF	G+ bacteria, G− bacteria	Lipid Bilayer	Mixed-αβ
Thanatin (8–21)	IIYCNRRTGKCQRM	G+ bacteria, G− bacteria, Fungus	Lipid Bilayer	β-sheet
50S ribosomal protein L1 HP (2–20) [Q16W]	AKKVFKRLEKLFSKIWNDK	G+ bacteria, G− bacteria, Fungus	Lipid Bilayer	β-sheet
Esculentin (1–21)	GIFSKLAGKKIKNLLISGLKG	G+ bacteria, G− bacteria, Cancer, Fungus, Mammalian Cell, Biofilm	Lipid Bilayer, DNA/RNA	β-sheet
Ovispirin-3 -OH	KNLRRIIRKIIHIIKKYG	G+ bacteria, G− bacteria, Cancer, Fungus, Mammalian Cell	Lipid Bilayer	β-sheet
Cecropin A (1–7) + Melittine (2–9), CM15	KWKLFKKIGAVLKVL	G+ bacteria, G− bacteria, Mammalian Cell, Biofilm	Lipid Bilayer	β-sheet
gp41w	KWASLWNWFNITNWLWYIK	G+ bacteria, G− bacteria, Mammalian Cell	Lipid Bilayer	Mixed-αβ
Anoplin [R5W]	GLLKWIKTLL	G+ bacteria, G− bacteria, Fungus, Mammalian Cell	Lipid Bilayer	β-sheet

The aforementioned data are sourced from DBAASP v3 [177], with the date of reference being February 2025.

Despite the significant potential of CAD, no current method has been able to generate AMPs with characteristics superior to those optimized using known natural peptides for training. This suggests that, during AMP optimization, multiple factors must be considered, including antimicrobial activity, cytotoxicity, resistance to protease degradation, and other biological functions. Furthermore, the generated AMP sequences should ideally surpass the training samples in functionality, while maintaining biological relevance and stability.

With continuous improvements in computational design methods and tools, the future of AMP design looks promising. It is expected that these approaches will allow for the creation of more precise and effective AMPs, ultimately driving their clinical application against a broad range of pathogens.

**Table 3:** Accessible databases for AMPs.

Database	URL
APD3	https://aps.unmc.edu/home (accessed on 20 March 2025)
Cybase	https://www.cybase.org.au/ (accessed on 20 March 2025)
BACTIBASE	http://gec.u-picardie.fr/adaptable/ (accessed on 20 March 2025)
PhytAMP	http://gec.u-picardie.fr/adaptable/ (accessed on 20 March 2025)
CAMP	https://camp.bicnirrh.res.in/ (accessed on 20 March 2025)
DADP	http://split4.pmfst.hr/dadp/ (accessed on 20 March 2025)
DBAASP v3	https://www.dbaasp.org/home (accessed on 20 March 2025)
DRAMP	http://dramp.cpu-bioinfor.org/ (accessed on 20 March 2025)

**Table 4:** Prediction tools for AMPs.

Prediction Tool	URL
APD3	https://aps.unmc.edu/home (accessed on 20 March 2025)
BAGEL	http://bagel.molgenrug.nl/ (accessed on 20 March 2025)
antiSMASH	https://antismash.secondarymetabolites.org/#!/start (accessed on 20 March 2025)
AMPA	https://tcoffee.crg.eu/apps/ampa/do (accessed on 20 March 2025)
AMP_Scanner	https://www.dveltri.com/ascan/ (accessed on 20 March 2025)
CyPred	http://biomine.cs.vcu.edu/servers/CyPred/ (accessed on 20 March 2025)
AVPpred	http://crdd.osdd.net/servers/avppred (accessed on 20 March 2025)
AntiBP3	https://webs.iiitd.edu.in/raghava/antibp3/ (accessed on 20 March 2025)

### 5.3. Conjugation of Antimicrobial Peptides

While AMPs are effective against a wide range of pathogens, their practical applications are often limited by issues such as structural instability and sensitivity to physiological conditions. One promising strategy to enhance the effectiveness of AMPs is the conjugation of AMPs with antibiotics or other molecules. This combination can improve the specificity of AMPs against target microorganisms while synergizing with antibiotics to reduce toxicity to host cells, ultimately enhancing therapeutic safety. By forming conjugates, researchers can address limitations such as poor solubility, low bioavailability, or the emergence of antibiotic resistance.

**Figure 5:**
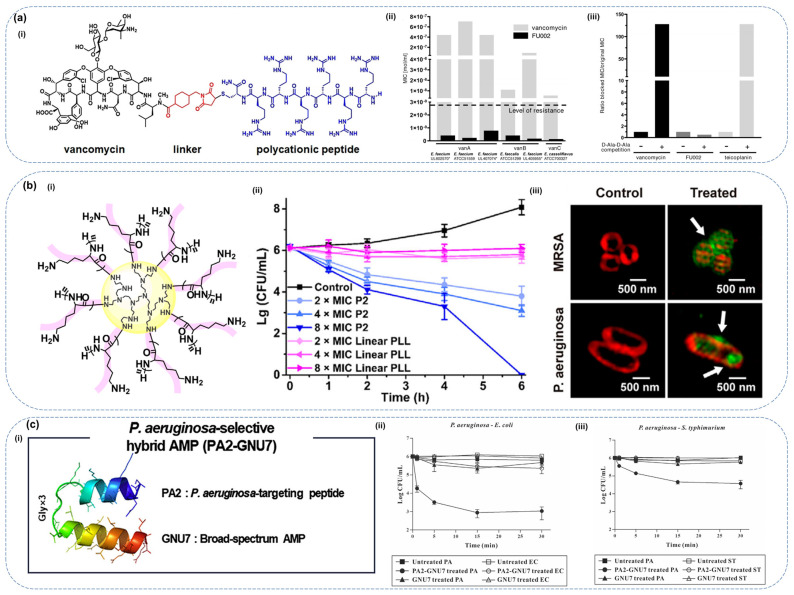
Strategies for enhancing the bactericidal activity of AMPs through conjugation with different molecules. (**a**) Conjugation of AMPs with antibiotics. (**i**) The peptide–antibiotic conjugate FU002, formed by conjugating an AMP with vancomycin. (**ii**) Antibacterial activity of vancomycin and its derivative FU002 (* = clinical isolate). (**iii**) Blocking experiments with the Nα,Nϵ-diacetyl-Lys-d-Ala-d-Ala binding motif on *Staphylococcus aureus* NCTC 10442. Reproduced with permission from ref. [178]. Copyright 2020, Wiley-VCH Verlag GmbH & Co. KGaA. (**b**) Conjugation of AMPs with polymers. (**i**) Schematic illustration of a star-shaped polymer (P2). (**ii**) Time-kill curves of P2 and linear PLL against MRSA. (**iii**) 3D-SIM images of bacteria before and after treatment with FITC-labeled P2 at 1 × MIC. Arrows indicate the local high green fluorescence on the membrane. Reproduced with permission from ref. [183]. Copyright 2019, Elsevier. (**c**) Conjugation of AMPs with peptides. (**i**) Schematic illustration of the hybrid peptide PA2-GUN7. (**ii**,**iii**) Selective bactericidal activity of the hybrid peptide against *P. aeruginosa*. Reproduced with permission from ref. [184]. Copyright 2020, Elsevier.

For instance, vancomycin, an antibiotic commonly used to treat multidrug-resistant infections caused by G+ bacteria, has faced growing resistance, particularly from vancomycin-resistant *Enterococcus faecium* (*E. faecium*) and *Staphylococcus aureus*. To overcome this challenge, Umstätter and co-authors developed a conjugate of vancomycin with polycationic peptide Hecate (Figure 5a). This conjugate (FU002) exhibited a remarkable 1000-fold increase in antimicrobial activity compared to the free antibiotic, while blockade experiments revealed that its bactericidal mechanism is distinctly different from those of vancomycin and teicoplanin. Meanwhile, it shows no cytotoxicity to mammalian cells, demonstrating its potential as an effective strategy to combat resistant pathogens [178]. Similarly, Ptaszyńska et al. conjugated an AMP HLopt with antibiotics levofloxacin and ciprofloxacin. While only the ciprofloxacin conjugate showed enhanced antimicrobial activity, both conjugates exhibited improved solubility over a wide pH range, unlike the parent antibiotics, which are soluble only at pH 5. This enhancement in solubility overcame the limitations of conventional drug-delivery systems, thus improving the bioavailability and therapeutic efficacy of the conjugated drugs [179]. In addition, the combination of GL13K and tobramycin can eradicate *P. aeruginosa* at concentrations where each drug alone is ineffective [180]. Additionally, ampicillin can enhance the killing of vancomycin-resistant Enterococcus (VRE) mediated by daptomycin and cationic host-defense peptides [181].

The combination of AMPs with antibiotics can also result in synergistic effects, where the antimicrobial activity of one compound is amplified by the other. A notable example is that the synergistic use of the AMP GL13K with the antibiotic tobramycin was found to effectively eradicate *P. aeruginosa* at concentrations where each drug alone would be ineffective. This demonstrates the potential of combining AMPs with antibiotics to combat resistant bacterial strains more effectively [180]. Additionally, ampicillin, when combined with daptomycin and cationic host-defense peptides, significantly enhanced the killing of VRE. This synergistic effect suggests that AMP–antibiotic conjugates could be a promising approach to treating difficult-to-treat infections caused by resistant strains [181].

These examples underscore the growing potential of AMP conjugation strategies, not only for improving antimicrobial efficacy but also for overcoming the challenges posed by antibiotic resistance and poor drug delivery. By leveraging the unique properties of both AMPs and antibiotics, such conjugates can provide a more versatile and effective approach to tackling multidrug-resistant bacterial infections.

In addition to conjugation with antibiotics, conjugating AMPs with polymers is also a highly effective approach, which can improve their stability, prolong their circulation time, and reduce toxicity. For instance, hydroxyapatite (HA) was conjugated with innate defense regulator-1018, forming the modified peptide SHABP, which showed enhanced antimicrobial activity against biofilm-associated microorganisms [182]. Moreover, Lu and colleagues designed a star-shaped molecular scaffold with polyethyleneimine as the core and L-lysine on the surface, which improved the stability of AMPs, protected them from proteolysis, and extended their duration of action in vivo (Figure 5b). Time-kill assays revealed that P2 exhibits significantly stronger bactericidal activity compared to PLL alone. These findings suggest that P2 promotes membrane-induced peptide aggregation and subsequent cellular internalization, potentially underpinning its enhanced antimicrobial efficacy [183].

Recently, researchers have focused on designing hybrid peptides by combining different AMPs or peptide segments, which can significantly enhance antimicrobial activity and targeting ability while reducing cytotoxicity. Researchers created a hybrid peptide PA2-GNU7 by combining the targeting peptide PA2, which binds to the OprF porin on *P. aeruginosa* with AMP GNU7. As shown in Figure 5c, PA2-GUN7 demonstrates remarkable selective bactericidal activity against *P. aeruginosa* [184]. In addition, a chimeric human defensin (H4), combining sequences from human β-defensin-3 (hBD-3) and human β-defensin-4 (hBD-4), showed superior antimicrobial activity compared to either hBD-3 or hBD-4 alone [185].

## 6. Challenges and Opportunities

The global antimicrobial resistance (AMR) crisis has prompted a surge of interest in alternative therapeutic agents, with AMPs emerging as a promising class of anti-infective agents. Due to their broad-spectrum activity, low likelihood of resistance development, and unique antibacterial mechanisms of action, AMPs present great potential in addressing infections caused by multidrug-resistant pathogens. However, despite their promising prospects, AMPs still face several significant challenges in clinical application.

One of the main challenges is toxicity, particularly nephrotoxicity and hemolysis. AMPs such as colistin have been reported to cause kidney damage at concentrations necessary for antimicrobial activity. This issue arises because the therapeutic concentrations of AMPs often overlap with the concentrations that induce acute kidney injury, narrowing the therapeutic window and limiting their clinical use [186,187,188]. Additionally, the positive charges of AMPs can interact with the negatively charged membranes of red blood cells, leading to hemolysis, which further limits their safety profile in vivo [189,190,191,192]. These toxicities raise concerns about the systemic use of AMPs, particularly for long-term or large-scale applications.

Another major challenge lies in the structural instability of many AMPs. Most natural AMPs are susceptible to degradation by proteases and have poor stability under physiological conditions, which affects their therapeutic efficacy. While various modifications, such as truncation, cyclization, and conjugation with other molecules, have been proposed to enhance AMP stability, finding a balance between maintaining high antibacterial activity and improving stability remains a critical issue.

In response to these challenges, several promising strategies are being explored. For instance, the design of AMP-based nanomedicines and biomaterial combinations offer new opportunities for improving the in vivo performance of AMPs. Encapsulation of AMPs in nanoparticles or conjugating them with polymers can improve their stability, enhance tissue penetration, and facilitate controlled release. These strategies help minimize toxicity while maximizing the therapeutic effects. Combining AMPs with biomaterials such as hydrogels or metal nanoparticles also holds great potential for improving their biocompatibility and enhancing their antibacterial properties. Furthermore, CAD tools such as APD3, dbAMP, and Ensemble-AMPPred have been invaluable in identifying novel AMPs and optimizing their structures. The integration of CAD technologies in AMP development has become a promising avenue for overcoming existing limitations. Using AMP databases and advanced machine learning algorithms, researchers can now predict the sequence-activity relationships of AMPs, thus enabling the design of peptides with enhanced antimicrobial activity and reduced toxicity.

While these advancements show significant promise, there are still several barriers to overcome, particularly in translating these findings from the laboratory to clinical practice. One major issue is the scalability of AMP production, especially when modified peptides or peptide–drug conjugates are involved. Achieving high-yield synthesis and maintaining consistency in the quality of the final product are essential for ensuring that AMPs can be produced at a commercial scale. Moreover, long-term stability remains a concern for AMP formulations, particularly in terms of storage, shelf-life, and resistance to proteolytic degradation in the body. Looking forward, personalized medicine could play a crucial role in the future of AMP-based therapies. By combining AMPs with genomic and microbiomic insights, treatments could be tailored to individual patients, optimizing both the efficacy and safety of AMP therapies. For example, AMPs could be selected based on a patient’s unique microbiome or immune profile, improving therapeutic outcomes and reducing adverse effects.

In summary, while AMPs hold tremendous potential as a new class of antimicrobial agents, they are still hindered by challenges related to toxicity, stability, and scalability. The future of AMP-based therapies will depend on continued innovations in biomaterials, nanotechnology, and computational design, as well as the successful integration of these peptides into clinically feasible formulations. The combination of AMPs with advanced drug-delivery systems and the use of personalized treatment approaches are likely to be the key to overcoming these obstacles. With these advancements, AMPs could play a pivotal role in the fight against antimicrobial resistance, providing a powerful alternative to traditional antibiotics.

## Data Availability

No new data were created or analyzed in this study. Data sharing is not applicable to this article.

## Abbreviations

The following abbreviations are used in this manuscript:

AMPs	antimicrobial peptides
NMR	nuclear magnetic resonance
CD	circular dichroism
MIC	minimum inhibitory concentration
PKA	protein kinase A
RhoG	Ras homolog family member G
LPS	lipopolysaccharide
FDA	Food and Drug Administration
PLGA	poly (lactic-co-glycolic acid)
PEG	polyethylene glycol
PVA	polyvinyl alcohol
CS	chitosan
MIC	minimal inhibit concentration
GRAS	generally recognized as safe
GMP	glycomacropeptide
MBC	minimum bactericidal concentration
LNPs	lipid nanoparticles
HSPC	L-α-phosphatidylcholine
DPPG	1,2-Dipalmitoyl-sn-glycero-3-phosphoglycerol
GMO	glyceryl monooleate
AZT	azithromycin
MRSA	methicillin-resistant *Staphylococcus aureus*
FBS	fetal bovine serum
PAO	plasma amine oxidase
ODEX	oxidized dextran
PRP	platelet-rich plasma
SBMA	sulfonate betaine ester
AAc	acrylic acid
MNPs	metal-based nanoparticles
AuNPs	gold nanoparticles
AgNPs	silver nanoparticles
OA1	Odorranain-A-OA1
CMCS/SA	chitosan/sodium alginate
R2AW	ranatuerin-2-AW
CAD	computer-aided design
SVM	Support Vector Machines
MDR	multidrug-resistant
VRE	vancomycin-resistant Enterococcus
HA	hydroxyapatite
hBD-3	β-defensin-3
hBD-4	β-defensin-4
PEI	polyethyleneimine
PLL	poly (L-lysine)
AMR	antimicrobial resistance
G+	Gram-positive
G−	Gram-negative
